# Transforming the health information system using mobile and geographic information technologies, Papua New Guinea

**DOI:** 10.2471/BLT.20.267823

**Published:** 2021-03-02

**Authors:** Alexander Rosewell, Phil Shearman, Sundar Ramamurthy, Rob Akers

**Affiliations:** a School of Population Health, University of New South Wales, Sydney 2052, Australia.; b Papua New Guinea Remote Sensing Centre, Port Moresby, Papua New Guinea.; c Asian Development Bank, Port Moresby, Papua New Guinea.

## Abstract

In the context of declining economic growth, now exacerbated by the coronavirus disease 2019 pandemic, Papua New Guinea is increasing the efficiency of its health systems to overcome difficulties in reaching global health and development targets. Before 2015, the national health information system was fragmented, underfunded, of limited utility and accessed infrequently by health authorities. We built an electronic system that integrated mobile technologies and geographic information system data sets of every house, village and health facility in the country. We piloted the system in 184 health facilities across five provinces between 2015 and 2016. By the end of 2020, the system’s mobile tablets were rolled out to 473 facilities in 13 provinces, while the online platform was available in health authorities of all 22 provinces, including church health services. Fractured data siloes of legacy health programmes have been integrated and a platform for civil registration systems established. We discuss how mobile technologies and geographic information systems have transformed health information systems in Papua New Guinea over the past 6 years by increasing the timeliness, completeness, quality, accessibility, flexibility, acceptability and utility of national health data. To achieve this transformation, we highlight the importance of considering the benefits of mobile tools and using rich geographic information systems data sets for health workers in primary care in addition to the needs of public health authorities.

## Résumé

Dans un contexte de déclin de la croissance économique, exacerbé par la pandémie de maladie à coronavirus, la Papouasie-Nouvelle-Guinée a décidé d'augmenter l'efficacité de ses systèmes sanitaires afin de surmonter les difficultés à atteindre les objectifs globaux en matière de santé et de développement. Avant 2015, le système d'information sanitaire national était fragmenté, sous-financé, peu utile et rarement consulté par les autorités sanitaires. Nous avons donc conçu un système électronique intégrant des technologies mobiles et des ensembles de données géographiques provenant de chaque ménage, de chaque village et de chaque établissement de soins du pays. Entre 2015 et 2016, nous avons piloté le système dans 184 établissements de soins répartis sur cinq provinces. Fin 2020, les tablettes mobiles du système ont été distribuées dans 473 établissements de 13 provinces, tandis que les autorités sanitaires des 22 provinces du pays, y compris les services sanitaires confessionnels, ont pu accéder à la plateforme en ligne. Les silos de données fragmentées des programmes de santé antérieurs y ont été incorporés et une plateforme destinée aux registres d'état civil a été créée. Le présent document se penche sur la manière dont les technologies d'information mobiles et géographiques ont transformé les systèmes d'information sanitaire en Papouasie-Nouvelle-Guinée ces six dernières années en améliorant la ponctualité, l'exhaustivité, la qualité, l'accessibilité, la flexibilité, la recevabilité et l'utilité des données nationales sur la santé. Pour réaliser cette transformation, il est à nos yeux essentiel de tenir compte des avantages que représentent les outils mobiles, et de tirer profit des vastes ensembles de données géographiques non seulement pour les travailleurs des soins de santé primaires, mais aussi pour les besoins des autorités de santé publique.

## Resumen

En el contexto de un crecimiento económico en declive, agravado ahora por la pandemia de la enfermedad por coronavirus, Papúa Nueva Guinea está aumentando la eficiencia de sus sistemas sanitarios para superar las dificultades para alcanzar los objetivos globales de salud y desarrollo. Antes de 2015, el sistema nacional de información sanitaria estaba fragmentado, carecía de fondos suficientes, su utilidad era limitada y las autoridades sanitarias accedían a él con poca frecuencia. Construimos un sistema electrónico que integraba tecnologías móviles y conjuntos de datos del sistema de información geográfica de cada casa, pueblo y centro de salud del país. Entre 2015 y 2016 pusimos a prueba el sistema en 184 centros de salud de cinco provincias. A finales de 2020, las tabletas móviles del sistema se implementaron en 473 centros de 13 provincias, mientras que la plataforma en línea estaba disponible en las autoridades sanitarias de las 22 provincias, incluidos los servicios de salud de las iglesias. Se han integrado los silos de datos fracturados de los programas sanitarios heredados y se ha establecido una plataforma para los sistemas de registro civil. Exponemos cómo las tecnologías móviles y los sistemas de información geográfica han transformado los sistemas de información sanitaria en Papúa Nueva Guinea en los últimos seis años, aumentando la puntualidad, la exhaustividad, la calidad, la accesibilidad, la flexibilidad, la aceptabilidad y la utilidad de los datos sanitarios nacionales. Para lograr esta transformación, destacamos la importancia de tener en cuenta los beneficios de las herramientas móviles y de utilizar conjuntos de datos ricos en sistemas de información geográfica para los trabajadores sanitarios de la atención primaria, además de las necesidades de las autoridades sanitarias públicas.

## ملخص

في ضوء النمو الاقتصادي المتراجع، والذي ازداد سوءًا الآن بسبب جائحة مرض فيروس كورونا، تعمل بابوا غينيا الجديدة على زيادة كفاءة نظمها الصحية للتغلب على الصعوبات في تحقيق الأهداف الصحية والتنموية العالمية. قبل عام 2015، كان النظام الوطني للمعلومات الصحية مجزأ ومحدود التمويل ويحقق فائدة محدودة ولا تدخل الهيئات الصحية عليه بانتظام. بنينا نظامًا إلكترونيًا يجمع بين تقنيات الأجهزة المحمولة ومجموعات بيانات نظام المعلومات الجغرافية لكل منزل وقرية ومنشأة صحية في البلد. جرّبنا النظام في 184 منشأة صحية على مستوى خمس مناطق بين عامي 2015 و2016. بحلول نهاية عام 2020، تم توزيع الأجهزة اللوحية المحمولة التابعة للنظام على 473 منشأة في 13 منطقة بينما توفرت المنصة عبر الإنترنت داخل الهيئات الصحية للمناطق كلها البالغ عددها 22 منطقة، تشمل الخدمات الصحية الكنسية. تم دمج مجمعات البيانات المجزأة الخاصة بالبرامج الصحية القديمة وتأسيس منصة لنظم التسجيل المدنية. لقد ناقشنا التحول الذي أدخلته تقنيات الأجهزة المحمولة ونظم المعلومات الجغرافية على نظم المعلومات الصحية في بابوا غينيا الجديدة على مدار السنوات الست الماضية من زيادة في الالتزام بالوقت والإنجاز والجودة والقدرة على الوصول إلى النظام والمرونة والتقبل والفائدة التي تحققها البيانات الصحية الوطنية. لتحقيق هذا التحول، نركز على أهمية النظر في الفوائد التي تحققها أدوات الأجهزة المحمولة واستخدام مجموعات البيانات الغنية على نظم المعلومات الجغرافية للعاملين الصحيين في الرعاية الأولية بالإضافة إلى احتياجات السلطات الصحية العامة.

## 摘要

在新冠肺炎疫情导致经济增长加速下滑的情况下，巴布亚新几内亚正在提高其卫生系统的效率，以克服在实现全球卫生和发展目标方面存在的困难。在 2015 年之前，国家卫生信息系统呈分散状态、资金不足且效用有限，卫生部门很少使用该系统。我们建立了一个电子系统，将移动技术与包含全国每个家庭、村庄和卫生设施信息的地理信息系统数据集相集成。2015 年至 2016 年，我们在 5 个省的 184 个卫生机构试运行了该系统。截至 2020 年底，已在 13 个省的 473 个机构处推出了该系统的移动设备和平板电脑版本，同时，22 个省的卫生部门（包括教会卫生服务机构）也均可通过在线平台使用该系统。整合了遗留卫生方案的零散数据竖井，并建立了一个民事登记系统平台。我们将讨论移动技术和地理信息系统在过去六年中如何通过提高国家卫生数据的及时性、完整性、质量、可获得性、灵活性、可接受性和实用性来改造巴布亚新几内亚的卫生信息系统。为了完成此类改造，我们强调，必须利用移动工具的优势，而且丰富的地理信息系统数据集除满足公共卫生部门的需求以外，还必须允许初级医护人员使用。

## Резюме

В ситуации снижения экономического роста, усугубленного к тому же пандемией коронавирусной инфекции, Папуа — Новая Гвинея наращивает эффективность систем здравоохранения ради преодоления сложностей в достижении глобальных целей в области здравоохранения и развития. До 2015 года национальная информационная система здравоохранения была разрозненной, испытывала недостаток финансирования, полезность ее была ограниченной и органы здравоохранения редко обращались к ней. Авторы создали электронную систему, которая объединила наборы систематических данных о местоположении каждого дома, поселка и учреждения здравоохранения в стране, полученные из геоинформационных систем, с технологиями мобильной связи. Пробный запуск системы был осуществлен в 184 учреждениях здравоохранения в период с 2015 по 2016 год и охватывал пять провинций. К концу 2020 года используемые в этой новой системе планшеты мобильной связи были переданы в 473 учреждения 13 провинций, а онлайн-платформа стала доступной в органах здравоохранения всех 22 провинций, включая услуги здравоохранения, оказываемые церковью. Разрозненные базы данных из устаревших программ здравоохранения были объединены, и была создана платформа для системы регистрации актов гражданского состояния. Авторы обсуждают тему того, каким образом мобильные технологии и геоинформационные системы преобразовали информационные системы здравоохранения в Папуа — Новой Гвинее в течение последних шести лет, расширив полноту данных и улучшив своевременность их предоставления, а также повысив качество, доступность, гибкость, приемлемость и полезность национальных данных в сфере здравоохранения. В рамках достижения этой трансформации авторы особенно отмечают важность преимуществ, которые предоставляет использование средств мобильной связи и широких наборов данных геоинформационных систем работникам системы первичной медико-санитарной помощи в дополнение к удовлетворению потребностей органов общественного здравоохранения.

## Introduction

Achievement of universal health coverage (UHC) and the sustainable development goals are currently unattainable in the fragile state of Papua New Guinea.^1^ Total health expenditure as a percentage of gross domestic product remains low in real terms and has been declining since 2004, while several key health access and quality indicators declined between 2006 and 2015. The World Bank has reported that donors provide about 20% of the country’s total annual spending on health but that the sources, amounts and recipients of funding are volatile.^2^ In the context of declining economic growth, development partners were urging the government of Papua New Guinea to increase the efficiency of current spending to create a stronger health system and better prepare itself for transition from Gavi, the Vaccine Alliance, and decreased vertical support from the Global Fund to Fight AIDS, Tuberculosis and Malaria.

Good health information is crucial for understanding and improving the efficiency of health-care delivery. Yet this core building-block of a robust health system is frequently absent in fragile settings. Despite donor partners’ investment of more than 182 million United States dollars in key disease programmes in Papua New Guinea before 2015,^3^ the national health information system was not benefiting to any extent. The system had become fractured, was difficult for users to access and was performing poorly.^4^ This deterioration meant that modelling, rather than disease reporting, was being used to understand the disease burden for priority disease programmes such as those for human immunodeficiency virus (HIV) control.^5^ Improvements to the country’s disparate, weak health information system for different health programmes and service-delivery levels was needed.

In this context, the Asian Development Bank’s National Health Services Sector Development Program conducted a pilot of mobile device technologies and geographic information systems in the capture and reporting of health data. Initially conducted in 184 health facilities in five provinces, the pilot was expanded following independent reviews in 2017–2018^6^ to become the national system in all 22 provinces. Mobile tablet devices for the electronic national health information system were supplied to 473 facilities across 13 provinces by the end of 2020. The electronic system aimed to repair the fractured health information system by integrating separate data collection systems into the one system to enable future disease-specific investments to benefit a larger number of health programmes. We describe the 6-year transformation of Papua New Guinea’s health information system over 2015–2020 and the implications for policy and practice.

## System description

Papua New Guinea’s electronic national health information system is based on a password-protected mobile application that provides an interface to the system (Box 1). Health-care workers access the system on tablet computers and the data provided feed into an online platform accessed by health authority staff. The application includes modules for data entry, automated summary data, a repository of national and international guidelines, a data dictionary and an automatically updating contact list. The frequency of user logins to the online platform is captured by the system.

Box 1 **Technical details of Papua New Guinea’s electronic national health information system**

Papua New Guinea’s electronic national health information system is based on a mobile device-based application that provides an interface to the system for health-care workers. We developed the application as an Android package kit that runs on the Android operating system (Google LLC, Mountain View, United States of America, USA). Data transmission to the server and synching between tablet devices and the server is done via second- (2G) and third-generation (3G) mobile telephone networks of both national mobile telecommunications providers. Application version updates are performed either directly by the end-user or remotely by the team managing the programme. We used a JavaScript platform as the interface, a standardized query language server for a back-end database, VMware (VMware Inc., Palo Alto, USA) as the back-end operating engine and a proprietary geographic information systems platform for the mapping interface. These choices are in accordance with the information communication technology standards of the national health authorities. Data storage on the in-country server also aligns with national standards for health information management, including confidentiality and redundancy. A helpdesk staffed by one person managed end-user issues during initial implementation of the system.

Monthly reporting covers outpatients, inpatients, well-baby services, immunizations, malaria, leprosy, HIV, tuberculosis, school health services, family planning, antenatal care, deliveries and drug shortages. The system also reports how many outreach clinics were planned and implemented and which programme staff from provincial authorities conducted supportive supervision. Every household, village and health facility in the country is geolocated within the system using geographic information systems overlaying high-resolution satellite imagery. The data captured by the system integrate with the geographic information system for analysis, visualization and reporting. Different types of health data have different levels of granularity; for example, tuberculosis is mapped to the house, malaria to the village or slum, vaccinations to the health facility. Individual patient data-captures enable the continuous updating of the geo-coded village list of more than 20 000 villages. Registered tuberculosis patients are now remotely geolocated to their household using searchable high-resolution satellite images. These data automatically feed into programme management tools, including dashboards, live maps and reports that automatically prioritize the required public health follow-up. To plan outreach services and campaigns, users can visualize and manually count every house in the country or in user-defined areas, using mapping tools that overlay onto high-resolution satellite images accurate to about 30 m.

## Improved data collection

Mobile technologies and geographic information systems are increasing the timeliness, completeness, quality, accessibility, flexibility, acceptability and utility of national health data in Papua New Guinea. 

### Accessibility

**Fig. 1:**
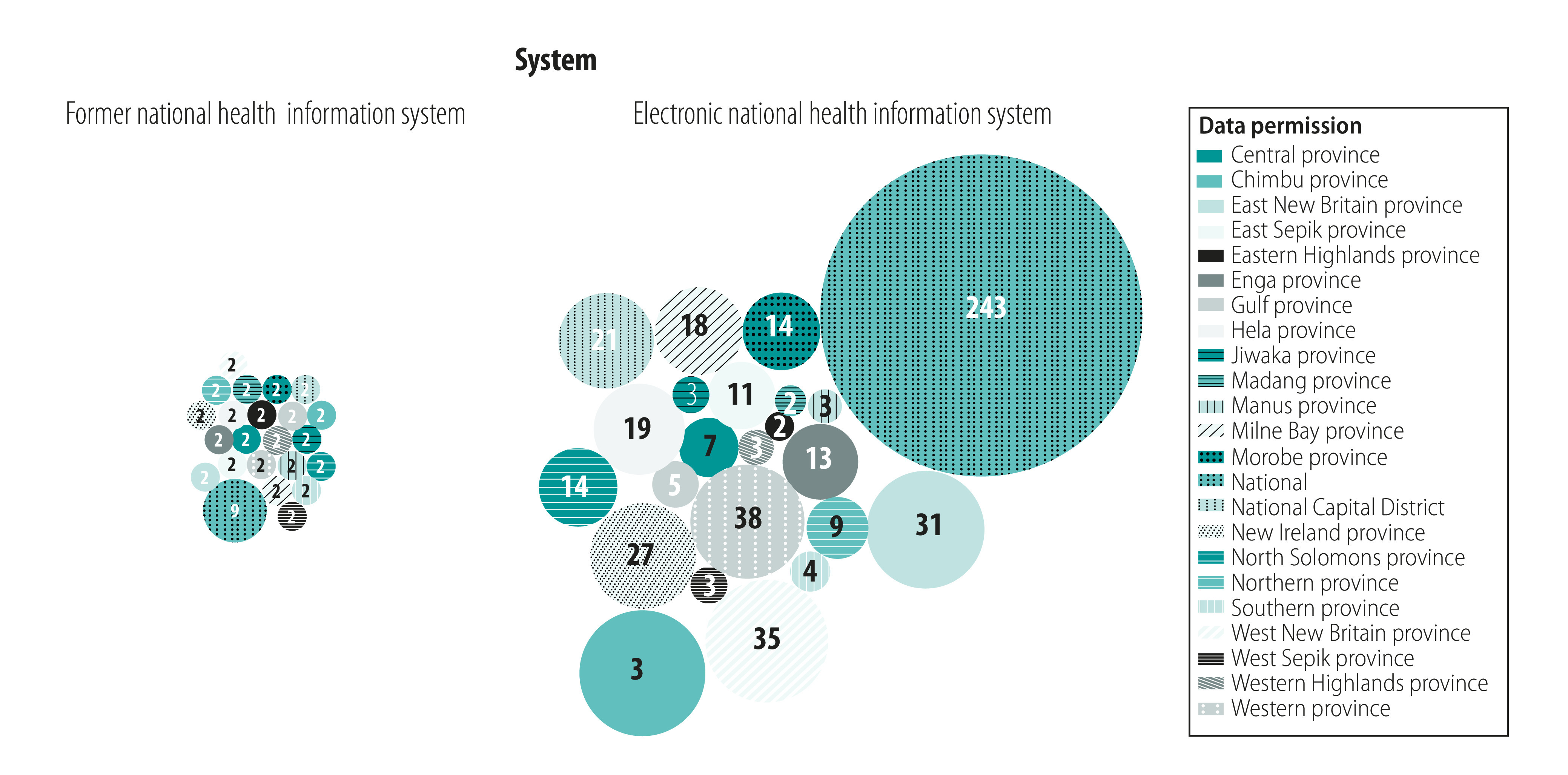
Health information system data availability in health authorities before and after transformation to an electronic system, Papua New Guinea

Note: The figure shows the number of staff in provincial and national health authorities who had access to data and reports in the old national health information system (left) and the new electronic system (right). Each circle is sized to the number of staff with system access. In general, staff in national health authorities, church services and partners are reported in the National category, while staff in provincial health authorities are under their province name.

Access to the old data platform was available to up to two health information staff in each province and a small team of about nine people in the national health authority. The new electronic information system has expanded user access to data and tools beyond these traditional users to programme managers and technical staff within national and subnational health authorities (Fig. 1). Faith-based health services provide the majority of primary health care in the country and previously had no access to their own data or analysis tools. The electronic system now enables church health service staff to access data and tools themselves, as illustrated by 556 logins to the new system in the past 12 months. Feedback to end-users has shifted from the manual production of one annual national paper report with an unclear distribution to automated monthly feedback to tablet devices in every reporting facility.

**Fig. 2:**
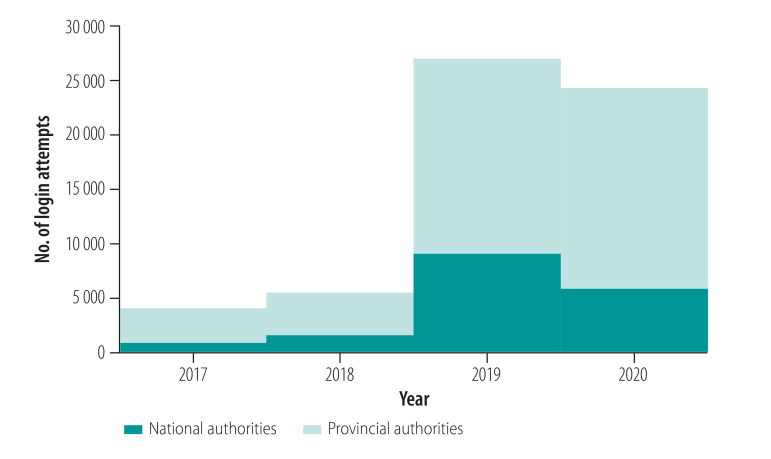
Logins to the electronic national health information system, by health authority, Papua New Guinea, 2017–2020

Note: The figure shows the number of times health authority staff logged into pages in the new electronic national health information system from 2017 to end of 2020, disaggregated by year and health authority permissions.

The new system tracks the adoption and use of health information across the various health programmes. The number of logins to the health information system by health authority staff and partners has risen from 4103 in 2017 to 24 334 in 2020 (Fig. 2). In 2019, there was a median of 101 monthly logins by health authorities and partners in provinces using mobile tablets versus 43 where data were entered from paper into the online portal within the provincial health authorities. The new system can therefore provide health authority management teams with a single metric on the degree to which programme staff at all levels engage with their programme data. The new system has important implications for public health practice through improving the efficiency of health programmes.

### Completeness

The electronic information system will help the government to better allocate its national health budget. Health authorities can now identify gaps in the completeness of national health data and the indicators used to inform health planning and budget allocation. For example, more than 4 years of data from the national tertiary referral hospital were missing from the national health information system, and service delivery data were not being captured by Mercy hospital ships and other mobile health-service providers. To highlight the implications using 2019 data, the national referral hospital’s inpatient caseload alone represented 17.5% (14 938) of the country’s 85 323 inpatients. These data would previously have been missing from the health indicators used for planning and budgeting.

### Timeliness

Monthly reporting from the electronic system is faster than before (10 versus 90 days), and enables near real-time use of hospital data, overcoming a 4-year delay in making these data available for use (Matheson D, et al., Asian Development Bank, Port Moresby, unpublished data, 2016). The increased timeliness of data has many benefits for public health practice, including that authorities and partners can monitor near real-time data from 473 clinical sites that were previously unavailable. In a country vulnerable to infectious disease outbreaks, this has important practice implications for outbreak detection and management, whereby events such as the nationwide outbreaks of circulating vaccine-derived poliovirus and coronavirus disease 2019 (COVID-19) were able to be monitored via the system.

### Quality

The Asian Development Bank’s independent review of the electronic national health information system found that the system’s data storage and automation of summary reports have likely reduced error in data collection across hundreds of sites (Matheson D, et al., Asian Development Bank, Port Moresby, unpublished data, 2016). In addition, data quality improvements only achievable at the point of electronic data entry would have strengthened the system’s data quality over the old national health information system. For example, the use of mandatory fields, mirrored fields, drop-down lists and rules on data formats in the data registers helps to optimize the internal validity of the data. Staff engagement with the system is enabling data-driven process improvement cycles. For example, tuberculosis staff in provincial health authorities are using mapping tools to identify individual tuberculosis patients who may require further care and are providing feedback to clinic staff on how to improve the timeliness and quality of tuberculosis data if anomalies across the data are identified.

## Case-based registers

We believe that an important element of the electronic information system’s success is balancing the burden of data entry with end-user benefits (such as automated reporting) in the context of the potential public health benefits of improved data capture. The large amount of complete and timely patient register data may be seen as a proxy for system acceptability by users and provides insights into how additional register data might be adopted (for example, to create an immunization register). 

Inpatient data entered into the system are automatically coded to the *International statistical classification of diseases and related health problems, 10th revision* (ICD-10) codes, with less than 1% of entries left to be coded by staff with higher levels of training. ICD-10 codes not available in the country’s shortlist can be remotely added without needing a version update to the mobile application. From 1 January 2015 to 31 December 2020, the electronic information system assisted health workers to report 706 261 inpatient discharges, ICD-coded and geolocated to village level.

The electronic system has revitalized platforms for civil registration systems which had previously stalled. By the end of 2020, 4881 births had been geolocated and registered and 44 188 ICD-10-coded inpatient deaths were geolocated to village level. The future integration of biometric data into the digital birth register and other case-based data sets should improve the efficiency of public health programmes by increasing the accuracy of the data (for example, knowing an individual requires which dose of a COVID-19 vaccine). The policy and practice implications of achieving a functioning civil registration system are important. National population estimates with local modifications are available in the electronic system, as is the potential for users to select alternate populations (for example, to produce population estimates for small areas) through the electronic national health information system in the future.

Malaria test and treatment records were previously only available in paper registers in health facilities (non-geolocated) or entered into a global donor-supported separate database then sent to the donor partner’s headquarters and not reported to the national system. The electronic information system has enabled the malaria programme to integrate these national data back into the national system so that users can analyse malaria records for all types of programme management needs; automatically run statistical algorithms over the data to identify outbreaks; detect shifts in non-malaria febrile illness; and provide the data required for malaria risk or incidence maps down to subdistrict level. By the end of 2020, 1 313 431 individual malaria patient test and treatment records, geo-coded to village level, had been reported (system described elsewhere).^6^

The electronic information system has also received 92 294 national HIV surveillance testing reports, 2241 treatment reports and 1953 risk-factor summaries over 2015–2020. However, national authorities, World Health Organization (WHO) and partners requested that the HIV programme data management remain unchanged and run separately outside the new system. In districts with extremely high HIV test-positivity but with easy access to health facilities, triangulation with monthly stock-out data (test kits, treatment) highlighted gaps in the policies and practices associated with the current model of support.

We have created a tuberculosis patient information management system within the electronic information system, currently being used in selected priority provinces of the country’s national tuberculosis emergency plan.^7^ The electronic system has replaced a poorly performing paper register and report-filing on spreadsheets,^8^ with detailed quarterly reports on drug-sensitive tuberculosis patients now being automated. The new system supports clinicians with the capture and ongoing management of geolocated tuberculosis patient information; automatically shifts patients between the intensive and continuation phase of therapy; automates patient progress summary reports and medical notes; provides clinicians with the drug, dose and number of pills to provide until the next appointment; sets dosage limits; and reports adverse events. Health authority users are provided with an ever-expanding suite of maps, reports and dashboards that help them improve the performance of the tuberculosis programme. Since 2019, 12 495 presumptive tuberculosis patients, 7527 drug-susceptible patients geolocated to household level and 29 drug-resistant patients were registered for care and public health management in the National Capital district alone.

The tuberculosis management system will also help to link patients with community support by generating automated geolocated job lists. The job lists will orient the actions of the hundreds of geolocated community health workers and treatment supporters who are paid to follow up on tuberculosis patients and their contacts. The new system also creates synergies for future patient registers to integrate the follow-up required of community workers (such as for children missing immunizations) with existing geolocated community tuberculosis job lists, to provide more comprehensive health system coverage. These are practical steps towards UHC.

## Benefits to clinicians

The electronic information system gives clinicians an incentive to enter timely, quality data, because they themselves benefit, in contrast to the previous system where clinicians knew their data may never be used by the health authorities. For example, the automation of more accurate monthly summary reports saves 2–3 days of clinician time in 473 facilities every month and avoids repeated data entry within provincial and national authorities (Matheson D, et al., Asian Development Bank, Port Moresby, unpublished data, 2016). The automated reporting in the tuberculosis patient information management system saves 2–3 days (for drug-sensitive tuberculosis) and 3–4 days (for drug-resistant tuberculosis) of clinician time per report. The system gives real-time guidance for managing individual tuberculosis patients (including maximal drug dosages) in a setting of limited training and frequent changes in complex clinical guidelines. The new system also gives support with patient scheduling linked to the amounts of tuberculosis medication to be provided; automated patient progress summaries; and reduced data entry by linking and mirroring data entry fields across registers. 

Further benefits for clinicians include automatically updating clinical and public health directories; two-way communication at no cost to staff; and improving the quality of data entry. Automated, real-time ICD coding removes the 3–4-year delay to using inpatient data. These improvements mean that the inpatient data set can now feed into outbreak detection processes using the disease outbreak dashboard to more quickly support clinicians when events are identified and to create automated monthly feedback bulletins. Future automated tools for clinicians and their communities could include mobile phone message reminders for immunizations and other appointments (with mobile numbers stored for potential communication during outreach and campaigns), and the linkage of digital personal identification systems to patient registers. National health authorities should now consider the integration of data on human resources, finances, drug supplies and other key data to broaden the scope of health system analyses and further increase efficiency.

## Successes and challenges

In Papua New Guinea, the health information system now feeds higher quality, timelier and more complete data into simple interactive dashboards, maps and operational tools. Platforms for stalled civil registration systems have been relaunched. Fractured data siloes have been integrated to assist future disease-specific investments in tools and innovations that also benefit non-funded programmes. Previously unavailable data sets such as malaria test and treatment records, geolocated to village level, are being collected. The real-time transmission of existing data sets is providing new and enhanced utility to the data. Challenges and opportunities to improve UHC are visualized through mapping service delivery and disease data down to the level of health facility, village and household. 

Factors in this success were focusing on building tools to benefit clinicians in primary care; leveraging the public–private partnership with a locally experienced implementing partner; harnessing mobile technologies that operate in 2G phone networks; and obtaining rich geographic information systems data sets. While the electronic information system platform has been a key part of the transformation, the public–private partnership with a company with proven record of accomplishment for innovation and political navigation in Papua New Guinea has also been important. Despite the many achievements, ensuring that health authority staff take full advantage of the new system remains an important challenge.

## Towards UHC

**Fig. 3:**
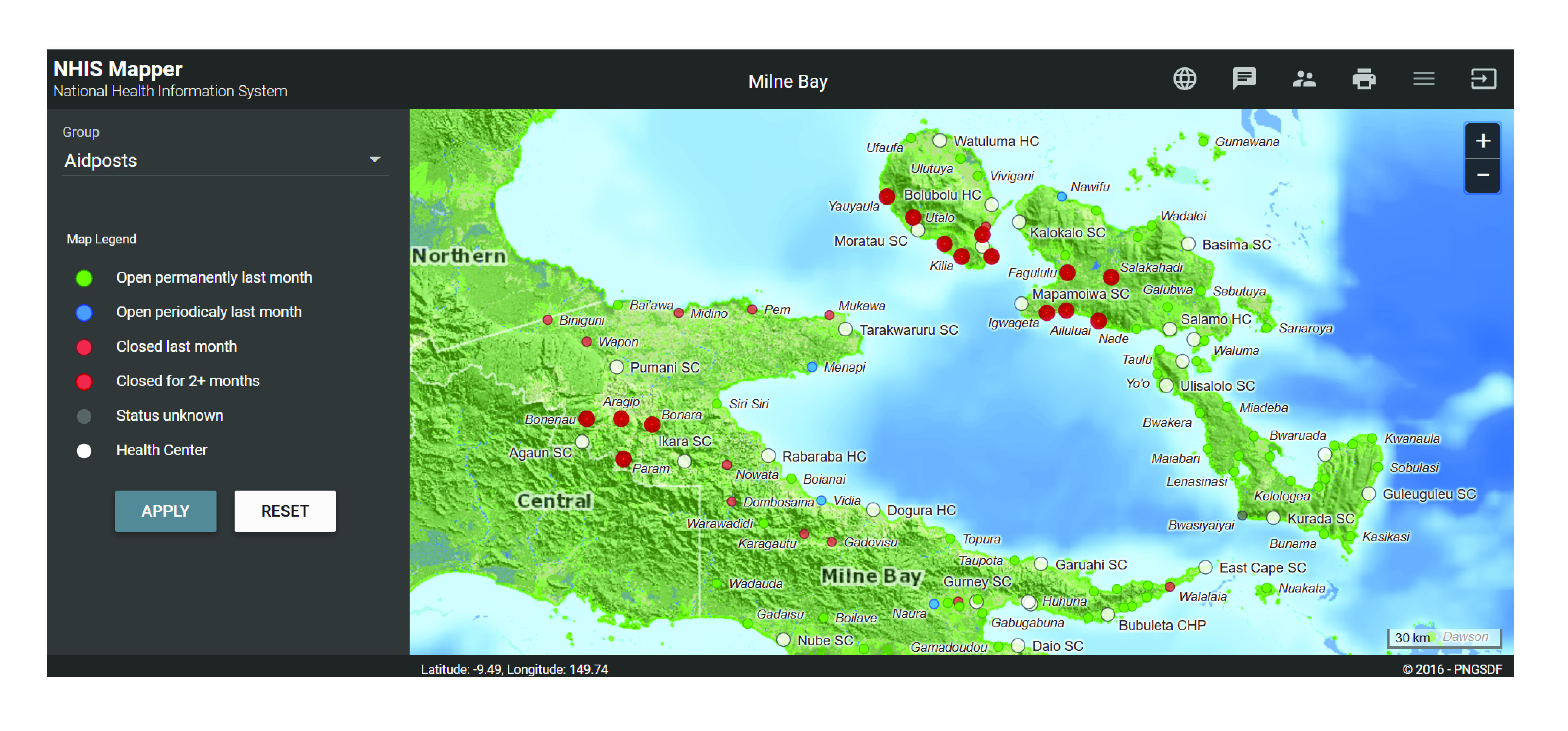
Example screenshot showing functional status of health facilities in the electronic national health information system, Papua New Guinea

CHP: community health post; HC: health centre; SC: sub-centre.

Note: The map is a screenshot of the user interface, displaying the location of health centres and aid posts. Aid posts are the first level of primary care and serve a population of 10 000 people. Papua New Guinea Remote Sensing Centre owns these maps and has provided a free licence to the National Department of Health for their use. These are the maps endorsed by the National Department of Health (Government of Papua New Guinea) for integration into the electronic national health information system. The geographic features of the map were derived from GeoSAR imagery. The locations of health centres were obtained by staff visiting these locations with hand-held global positioning system units.

Better information about the quantity, distribution and growth of populations is crucial to sustainable development globally.^9^ The importance of integrating these data into service delivery and performance measurement is clearly outlined in the investments of Gavi^10^ and the Bill & Melinda Gates Foundation,^11^ particularly for hard-to-reach urban slum populations. According to the WHO Director-General, “all roads lead to universal health coverage – the top priority at WHO”.^12^ However, without knowing where facilities are located and if they are open or closed (Fig. 3; available at: http://www.who.int/bulletin/volumes/99/5/20-267823), where the population resides and the villages where services reach and do not reach, a country will not be on the road to UHC.

Visualizing the geographical distribution of disease and service delivery performance using real-time health information system data alongside where the population actually resides can enable planners and decision-makers to better allocate resources. For example, the new information system in Papua New Guinea makes it possible to better understand UHC issues such as zero-dose communities (children who never received a first dose of a diphtheria–tetanus–pertussis-containing vaccine). Future geographic information innovations within the system may include: recommendations to health workers on which communities should be targeted for outreach services; capture of geo-validated outreach delivery data; and automating the individual tasking of community workers to follow up on patients lost to interventions. Given the problematic nature of census data in Papua New Guinea,^13^ house counts based on geographic information systems and household composition data could be used to generate small-area population estimates.^14^ These local data have the potential to further improve efficiencies in health service delivery.

Papua New Guinea continues to transform its health information system with better quality and analysis of data being used to save lives more efficiently and effectively. Despite the achievements of the electronic national health information system across 473 sites in challenging settings, many obstacles remain on the road to UHC in Papua New Guinea. National health authorities must continue to strengthen staff engagement with the system to improve the lives of its citizens and must effectively align donor support to optimize impact for all programmes.
